# Evaluation of nurse-led follow up for patients undergoing pelvic radiotherapy

**DOI:** 10.1054/bjoc.2001.2173

**Published:** 2001-12

**Authors:** S Faithfull, J Corner, L Meyer, R Huddart, D Dearnaley

**Affiliations:** Centre for Cancer and Palliative Care Studies, Institute of Cancer Research, 15 Cotswold Road, Sutton, Surrey; Centre for Cancer and Palliative Care Studies, Institute of Cancer Research, Royal Marsden Hospital Trust, Fulham Road, London; Clinical Trials Office, Institute of Cancer Research, Sutton, UK

**Keywords:** radiation, acute side effects, supportive care, nurse-led clinics

## Abstract

This study reports results from a randomised controlled trial of nurse-led care and was designed to determine whether nurse-led follow up improved patients morbidity and satisfaction with care in men treated with radical radiotherapy for prostate and bladder cancer. The aim was to compare outcomes in terms of toxicity, symptoms experienced, quality of life, satisfaction with care and health care costs, between those receiving nurse-led care and a group receiving standard care. The study population was of men prescribed radical radiotherapy (greater than 60 Gy). Participants completed self-assessment questionnaires for symptoms and quality of life within the first week of radiotherapy treatment, at week 3, 6 and 12 weeks from start of radiotherapy. Satisfaction with clinical care was also assessed at 12 weeks post-treatment. Observer-rated RTOG toxicity scores were recorded pre-treatment, weeks 1, 3, 6 and 12 weeks from start of radiotherapy. The results presented in this paper are on 115 of 132 (87%) of eligible men who agreed to enter the randomised trial. 6 men (4%) refused and 11 (8%) were missed for inclusion in the study. Data were analysed as a comparison at cross-sectional time points and as a general linear model using multiple regression. There was no significant difference in maximum symptom scores over the time of the trial between nurse-led follow-up care and conventional medical care. Differences were seen in scores in the initial self assessment of symptoms (week 1) that may have been as a result of early nursing intervention. Those men who had received nurse-led care were significantly more satisfied (*P* < 0.002) at 12 weeks and valued the continuity of the service provided. There were also significant (*P* < 0.001) cost benefits, with a 31% reduction in costs with nurse-led, compared to medically led care. Evidence from this study suggests that a specialist nurse is able to provide safe follow up for men undergoing radiotherapy. The intervention focused on coping with symptoms, and provided continuity of care and telephone support. Further work is required to improve the management of patients during and after radiotherapy. © 2001 Cancer Research Campaign http://www.bjcancer.com


*British Journal of Cancer* (2001) **85**(12), 1853–1864
© 2001 Cancer Research Campaign
doi: 10.1054/ bjoc.2001.2173, available online at http://www.idealibrary.com on IDEAL

http://www.bjcancer.com

### Evaluation of nurse-led follow up for patients undergoing pelvic radiotherapy

**S Faithfull[1], J Corner[2], L Meyer[3], R Huddart[2] and D Dearnaley[2]**

[1]Centre for Cancer and Palliative Care Studies, Institute of Cancer Research, 15 Cotswold Road, Sutton, Surrey; [2]Centre for Cancer and Palliative Care Studies, Institute of Cancer Research, Royal Marsden Hospital, Trust, Fulham Road, London; [3]Clinical Trials Office, Institute of Cancer Research, Sutton, UK

**Summary** This study reports results from a randomised controlled trial of nurse-led care and was designed to determine whether nurse-led follow up improved patients morbidity and satisfaction with care in men treated with radical radiotherapy for prostate and bladder cancer. The aim was to compare outcomes in terms of toxicity, symptoms experienced, quality of life, satisfaction with care and health care costs, between those receiving nurse-led care and a group receiving standard care. The study population was of men prescribed radical radiotherapy (greater than 60 Gy). Participants completed self-assessment questionnaires for symptoms and quality of life within the first week of radiotherapy treatment, at week 3, 6 and 12 weeks from start of radiotherapy. Satisfaction with clinical care was also assessed at 12 weeks post-treatment. Observer-rated RTOG toxicity scores were recorded pre-treatment, weeks 1, 3, 6 and 12 weeks from start of radiotherapy. The results presented in this paper are on 115 of 132 (87%) of eligible men who agreed to enter the randomised trial. 6 men (4%) refused and 11 (8%) were missed for inclusion in the study. Data were analysed as a comparison at cross-sectional time points and as a general linear model using multiple regression. There was no significant difference in maximum symptom scores over the time of the trial between nurse-led follow-up care and conventional medical care. Differences were seen in scores in the initial self assessment of symptoms (week 1) that may have been as a result of early nursing intervention. Those men who had received nurse-led care were significantly more satisfied ($P < 0.002$) at 12 weeks and valued the continuity of the service provided. There were also significant ($P < 0.001$) cost benefits, with a 31% reduction in costs with nurse-led, compared to medically led care. Evidence from this study suggests that a specialist nurse is able to provide safe follow up for men undergoing radiotherapy. The intervention focused on coping with symptoms, and provided continuity of care and telephone support. Further work is required to improve the management of patients during and after radiotherapy. © 2001 Cancer Research Campaign http://www.bjcancer.com

**Keywords**: radiation; acute side effects; supportive care; nurse-led clinics

Radiotherapy plays a key role in the treatment of cancer with over 50% of individuals now receiving radiation at some time during the course of their disease. The increasing number of men requiring radiotherapy impacts on supportive services such as follow-up care. Current management of radiotherapy is focused on treatment provided on an outpatient basis. Monitoring for side effects is at regular clinic visits, when there may be little opportunity for discussion of the impact of treatment (Rotman et al, 1977; Strohl, 1988). Traditional approaches to the monitoring of radiotherapy treatment focus primarily on physical symptoms and response to treatment (Steinberg and Rose, 1996). Questions are now arising as to the effectiveness of these services, especially when economic restraints and growing patient numbers put traditional patterns of care under pressure.

It has been suggested that specialist nurses have the potential to play a central role in identifying symptoms of disease, adverse effects of radiotherapy, clinical management and in promoting health. Brown and Grimes' (1993) meta analysis of nurse practitioner care, found that specialist nursing was more focused on promoting health than standard medical care which resulted in increased satisfaction with care and was cost effective. Richardson and Maynard (1995) in an economic review of the possibility of exchanging medical and nursing roles, contested these findings and were less certain that these nurse practitioner roles could be implemented in the United Kingdom. They cite the increased education level of nurses in the USA and argue that these roles would be difficult to apply in the National Health Service. However, a growing number of studies in the UK are demonstrating the value that such specialist nursing can provide (Ridsdale et al, 1997).

In radiotherapy, attempts have been made to study how specialist nursing can be implemented. Norcross Weintraub (1990) performed a randomised trial in the USA comparing health education or the provision of specialist nursing against conventional care. They suggested advantages for the role of the radiotherapy nurse, but this study was under-powered and unable to provide statistical evidence of these benefits. A descriptive study in the UK of the provision of a specialist nurse for those undergoing cranial irradiation found a subsequent 30% reduction in medical workload (James et al, 1994). A more recent observational study found that specialist nurses within a radiotherapy setting provided greater interaction and initiated more supportive care than there medical colleagues. The authors suggest that this model of care may provide more effective support for patients undergoing radiotherapy (Campbell et al, 2000). However, few of these studies have observed patient outcomes or explored the economic implications of such a nurse-led service.

The issue of utilising specialist nurses, as a substitute for medical care, is topical but rarely evaluated. Evaluation of a

*Received 24 June 2001*
*Accepted 18 September 2001*

*Correspondence to:* S Faithfull service should reflect not only utilisation, but also the impact on patients. We evaluated the effectiveness of nurse-led follow up vs. conventional medical care and investigated how these services managed symptoms and patients' quality of life. Overall evaluation of the service included satisfaction with health care and economic costs. The hypothesis was that the provision of nurse-led care would reduce side effects of therapy and improve quality of life for men undergoing pelvic radiotherapy. A secondary hypothesis was that nurse-led care would increase patients' satisfaction and reduce costs.

### METHODS

Men included in the study were those undergoing radical (greater than 60 Gy) radiotherapy for prostate or bladder cancer. They were randomised either to a group receiving conventional care (control), or to a group receiving instead care from a clinical nurse specialist (intervention). Both groups had toxicitys from treatment monitored and their quality of life and perception of symptom severity assessed. Randomisation was carried out by the Clinical Trials Unit, Institute of Cancer Research, Sutton, and was stratified to provide a balanced representation of men with prostate and bladder cancer in the 2 randomised groups. Men were approached for consent in the planning stages of radiotherapy and asked to participate in the trial at start of radiotherapy. Out of the 131 of those eligible, 6 men refused, not wishing to have nurse-led care and 11 were missed due to starting radiotherapy prior to the researcher being able to access them. In total 115 (85%) of those undergoing radical treatment were involved in the study.

Radiotherapy was CT (computerised tomography) planned and delivered using 5–10 MeV linear accelerators, generally using 3 field techniques with anterior and lateral or posterior oblique fields. For men with prostate cancer the target volume was the prostate (± seminal vesicles) treated with a 1.0–1.5 cm margin. Conformal radiotherapy methods and treatment delivery using a multileaf collimator was used for 25 men treated in a dose-escalation study. The remaining patients were treated with conventionally collimated fields. All radiotherapy fields were treated daily using conventional 2 Gy fractions to give a total dose of 64–74 Gy prescribed to the isocentre. For patients with bladder cancer, the whole bladder or tumour was treated with a 1.5–2 cm margin using conventional radiotherapy methods and 2 Gy daily fractions to a total dose of 60–64 Gy. Both the control and intervention groups were assessed within the first week of starting radiotherapy, week 3, 6 and at 12 weeks following start of radiotherapy (Figure 1). It was estimated that 164 patients would be required to detect an absolute difference of 20% in morbidity between the 2 groups of the study ($\alpha = 0.05$, power 80%). Morbidity was defined as a difference of 20% in severity of urinary symptoms and decreased global quality of life at 6 weeks, when acute radiotherapy side effects would be expected to be most pronounced.

Accrual to the study was lower than anticipated for 2 reasons. Firstly, fewer men than expected were receiving radical radiotherapy for bladder cancer. Secondly, although a second centre was expected to participate it did not in the end enter patients. In consequence the data were reviewed after one year by an independent data monitoring committee (DMC). The reduced accrual resulted in a predicted shortfall of 40 patients. On the advice of the DMC, the study was closed to recruitment and follow up of existing subjects continued.

### Assessment of symptoms and quality of life

Data were collected longitudinally with observer-rated toxicity scores and self-assessment of symptoms, quality of life, experience and satisfaction with care measures. The RTOG/EORTC (Cox et al, 1995) observer-rated toxicity scale provided a conventional measure with which to compare the acute toxicity of symptoms between the 2 groups and was completed pre-treatment and at 1, 3, 6 and 12 weeks from start of radiotherapy. Both the specialist nurse and physicians undertaking clinics collected data. In order to define inter-observer variation a subgroup of patients ($n = 49$) was assessed independently from the 2 clinics by another clinician and, out of 490 total observations, there was a 96% agreement in scores. Self-assessment of symptoms was by using a 13-item questionnaire with 100 mm visual analogue scales, which monitored not only the occurrence and severity, but also the distress, and more global impact that these symptoms had on daily activities. This self-assessment questionnaire was developed from semi-structured interviews in a similar patient group (Faithfull, 1995). Participants completed this assessment during their first week of radiotherapy and after 3, 6 and 12 weeks. The EORTC QLQ-C30 (Aaronson et al, 1993) was used to explore the individuals' psychosocial adjustment to their illness and perceived severity of physical symptoms. The questionnaire covered several areas of quality of life, the physical, emotional, cognitive, social and role functioning effects as well as global quality of life. This was completed within the first week of starting radiotherapy, week 6 and 12.

### Assessment of satisfaction and economic costs

Overall satisfaction with the clinical care was evaluated using a self-assessment questionnaire. This was given to participants 12 weeks from start of radiotherapy ($n = 115$) and requested to be returned anonymously by post ($n = 108$). This tool was based on the Newcastle satisfaction with nursing scale (Thomas and MacMillan, 1995) which was developed by eliciting patients' concepts of good and unsatisfactory care. The questionnaire was designed to identify and discriminate between different types of ward organisation and it has been shown to be valid and reliable in this context. The questionnaire explored domains such as information provision, interpersonal skills and communication, continuity of care, symptom management and awareness of patient needs. For this study it was adapted to reflect a more general interpretation of health care organisation from an outpatient basis and therefore results should be interpreted in this context. Changes to the questionnaire were to replace nurse with doctor/nurse in all questions and remove 14 specific inpatient enquiries such as night care experience. A further 9 questions were removed in seeking patients opinions of health care as they focused on ward-based health-care activity. Costs were based on the hospital accounting system and prescription records, drug costs were standardised from the date of analysis so that comparisons could be made longitudinally (Yates, 1998). Health-care costs were analysed in units. Separate unit costs were calculated for medications, microbiology investigations and services utilised during the 3 months a participant was part of the study. Service utilisation included relevant salaries, including nursing and medical salaries, equipment costs and rental of accommodation. Patient costs such as time off work or travel to the hospital, were not included. The costs of radiotherapy treatment were also not included as these were relatively

 *© 2001 Cancer Research Campaign*

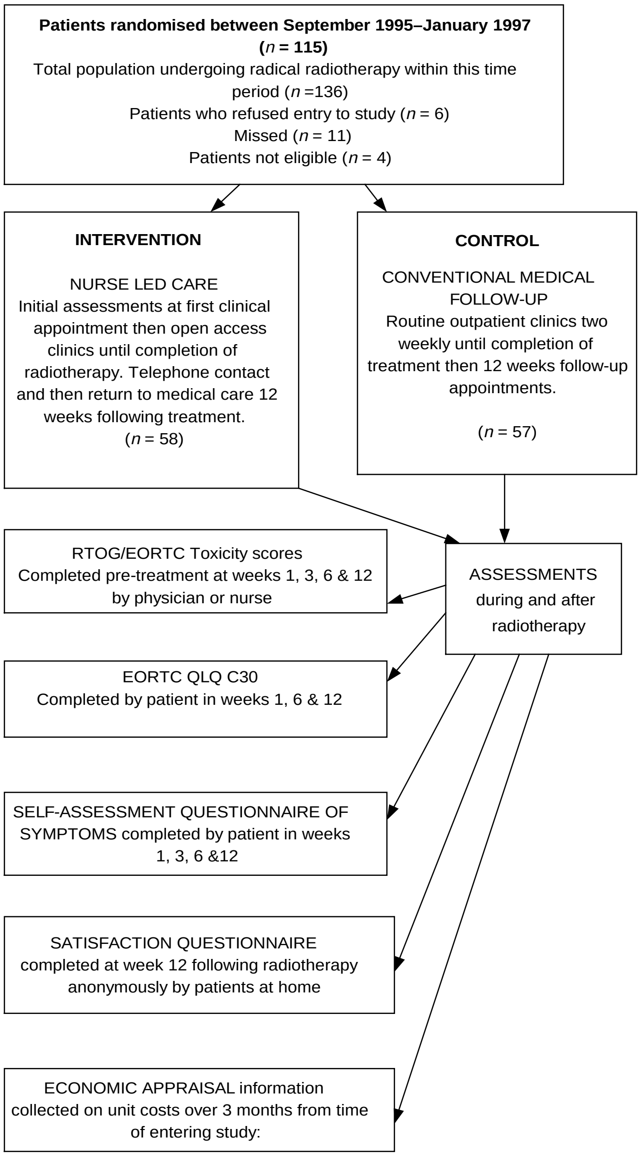

**Figure 1** Design of randomised controlled trial of nurse-led care v standard medical care in patients with prostate and bladder cancer undergoing pelvic radiotherapy constant. Elements of care that were monitored were admissions to hospital, either as a day case or for overnight stay, outpatient appointments and any additional services such as visits to the radiotherapy nurse. The economic costs should be reviewed as a unit of comparison rather than the 'real' costs of follow-up care from radiotherapy.

### Intervention and control approach

The nurse-led care (intervention) provided a separate service specifically for patients within the study. Contact was established at start of therapy and was continued throughout treatment until 12 weeks from start of radiotherapy, when patients returned to medical care. The specialist nurse at initial contact provided information and answered patient questions. The nurse also provided men and their families with leaflets on healthy eating, radiotherapy and how to manage urinary symptoms during radiotherapy. Attendance at the nurse-led clinic was organised for within the first week and last week of radiotherapy. Further appointments could be negotiated as required. Telephone contact was maintained between clinic appointments to assess health status. Nurse-led outpatients appointments were for 20 minutes. The intervention approach was based on exploring the individual's understanding of their cancer diagnosis, symptoms and the meaning of the illness. The provision of information and practical advice on how to recognise early symptoms, what to expect from treatment and how to manage existing problems were considered. A protocol of medication and management for symptoms was agreed with the responsible consultants (Figure 2).

Conventional care (control) consisted of routine medical appointments lasting 10 minutes within the urology outpatient setting. These were arranged routinely at start of radiotherapy treatment at either weekly (for men with bladder cancer) or 2-weekly (for those with prostate cancer) intervals. These appointments continued throughout the duration of therapy. This was a consultant-led clinic with a group of 6 physicians, 2 of whom were

*© 2001 Cancer Research Campaign*

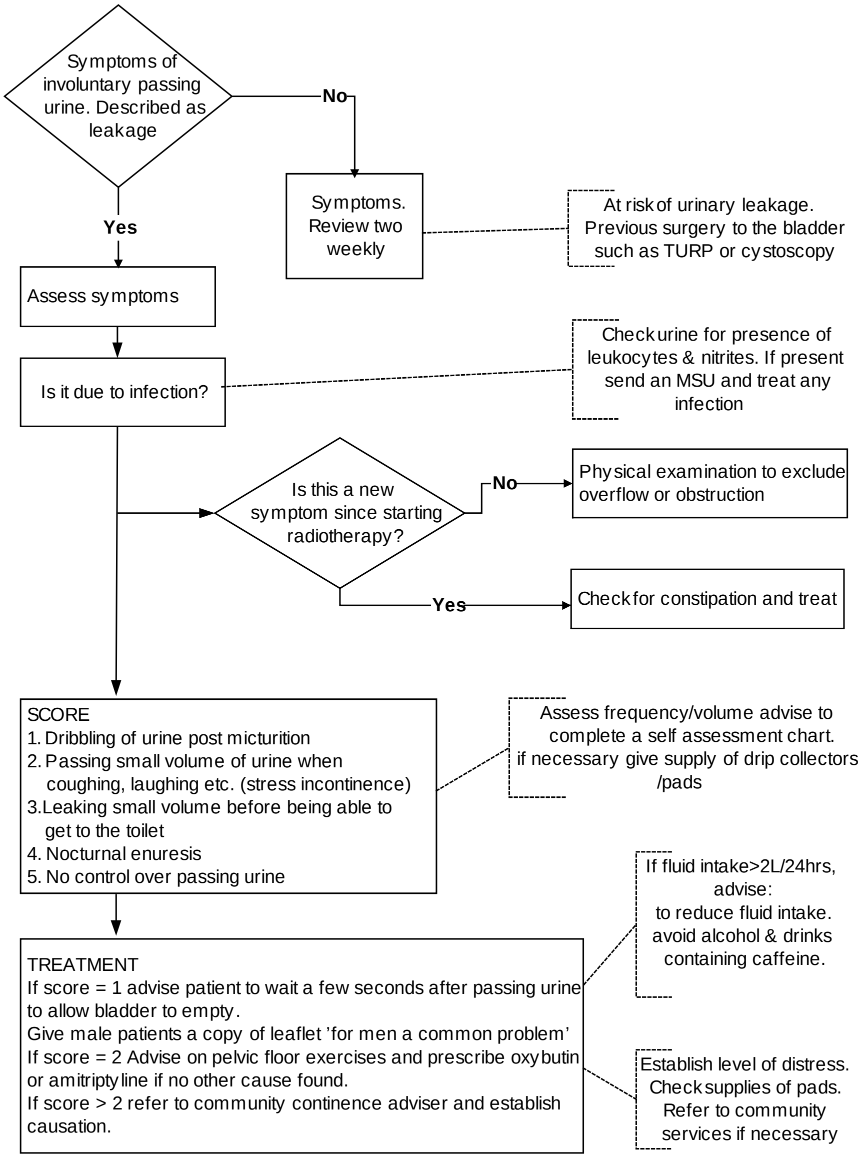

**Figure 2** Example of algorithm of protocol for the management of urinary incontinence pre-fellowship registrars, 2 post-fellowship registrars and 2 medical research fellows. Medical care consisted of assessment of toxicity, symptom management and monitoring of progress through treatment. Following treatment patients returned to clinic 12 weeks from start of radiotherapy.

Both intervention and control group had access to outpatient nurses and a radiotherapy nurse based within the treatment suite. Auditing of radiotherapy treatment planning and delivery was assessed weekly within a multi-disciplinary team for all patients in the trial.

### Statistical methods

Data were analysed in 3 ways firstly by comparing data at cross-sectional time points at week 1, 3, 6 and 12 from start of radiotherapy, secondly using self-assessed symptoms as a general linear model over the time of radiotherapy and thirdly evaluating the health-care costs and satisfaction with care. This provided data that compared data at key time points as well as across the treatment trajectory. For self assessment of symptoms an algorithm was used that transformed scores for the individual items that made up each symptom which determined the total symptom scores (Mathews et al, 1990). This gave a composite score potentially ranging from 0–100 that included the severity, occurrence and distress of that symptom. As can be expected with quality of life scores, data were not normally distributed and non-parametric significance tests were carried out, generally based on the Mann–Whitney U test. RTOG scores were assessed for association between randomised groups using $\chi^2$ tests. Toxicity scores were dichotomised into low-toxicity RTOG 1 and 2 scores or severe toxicity which included RTOG scores greater than 2. These scores reflected maximum change in toxicity from pre-radiotherapy scores.

Secondly multivariate analysis was conducted to explore how randomisation, demographic and treatment factors influenced symptom severity. Men were coded as having high or low symptom scores at 6 weeks according to whether they fell above or below the median score. These codes formed the basis of the multiple regression analysis. Thirdly satisfaction scores collected

 *© 2001 Cancer Research Campaign*

at 12 weeks were based on an algorithm score comparing the experience and satisfaction with care (Thomas and MacMillan, 1995). Questions were first re-coded to reflect scores from 0–6, the questions were then summed and divided by the number of validly answered scores within that question. This was then divided by 6 (number of codes) and multiplied by 100 to give a global score where 100 represented the best possible experience. Economic costs were gathered through patient records and hospital data base, these were summed and analysed based on the Mann–Whitney U test.

The compliance rate for completion of questionnaires was variable across the time points with fewer assessments being completed later on during follow-up, this resulted in missing data. This non-response was partly as a result of a reliance on questionnaires being distributed during outpatient clinics, so patients may not have received assessments and possibly assessment fatigue. Self-assessed symptom data that was completed was included in the analysis despite missing data at some time points. Data were analysed on a symptom-by-symptom analysis and if data were missing from an element of that visual analogue scale this question was excluded from the patient's analysis. Quality of life data also diminished over time partly for the same reason and partially completed questionnaires were not considered in the analysis. RTOG data were less of a problem as these were objective data scored at the time of patient visits, there were therefore no missing data in this data set. Partially completed satisfaction data were addressed by adjustment of the algorithm to exclude the missing data scores.

### RESULTS

### Patient characteristics

Demographic data for all randomised patients are shown in Table 1. There were no statistically significant differences in treatment, stage of disease or patient characteristics between control and intervention groups. Most of the men were elderly with a median age of 70 and an age range from 40–83. This age was reflected in employment history with over half of the population being retired. The sample consisted mainly of men undergoing prostate cancer treatment (83%) and a smaller proportion of men (17%) undergoing pelvic radiotherapy for bladder cancer.

Questionnaire compliance was high initially (96%) but there was a decrease in completion of questionnaires over time (88% at week 6). There were also differences in rates of completion for different questionnaires, for example at the 12-week time point 94% of satisfaction questionnaires and 81% of the EORTC quality-of-life questionnaires were returned.

### Process of the clinics

In the intervention group it was planned that patients returned to medical care at 12 weeks. This joint medical and nursing appointment was counted as part of the intervention within the nursing costs. However, 37 of the intervention patients had more than the one planned medical appointment. 15 men had 2 medical appointments and 2 patients were seen on multiple occasions (7 clinic appointments). The reasons for these extra medical appointments were that these men had a medical problem needing prescriptions not included in the protocol (Figure 2). Those men requiring multiple appointments had co-morbid disease that required monitoring and prescriptions (diabetes and heart disease) and were therefore seen by the nurse within the medical clinic. The mean number of clinic appointments for the intervention group was 3.1 (range 1–8) compared to 5.8 (range 3–11) for the control group. In the intervention group men received a total of 73.3 hours of consultation time (a mean of 1.2 hours per patient) where as in the control group men received 58.3 hours in total (a mean of 1.0 hours per patient). If the additional appointments with the radiotherapy nurse are included this brings the total consulation time with health carers to 96.3 hours for men in the intervention group and 93.9 hours in the control group (mean 1.6 consultation hours

**Table 1** Demographic characteristics of patients taking part in the study

| | **Control (*n* = 57)** | **Intervention (*n* = 58)** | **Total (*n* = 115)** |
|---|---|---|---|
| Age median (range) | 70 (49–83) | 70 (51–80) | 70 (49–83) |
| Diagnosis and stage | | | |
| Adenocarcinoma of prostate | 47 (82%) | 48 (83%) | 95 (83%) |
| T1 | 5 (11%) | 4 (8%) | 9 (9%) |
| T2 | 16 (34%) | 17 (38%) | 33 (35%) |
| T3 | 23 (49%) | 27 (56%) | 40 (53%) |
| T4 | 3 (6%) | 0 (0%) | 3 (3%) |
| Transitional cell cancer of the bladder | 10 (18%) | 10 (17%) | 20 (17%) |
| T1 | 0 (0%) | 0 (0%) | 0 (0%) |
| T2 | 3 (30%) | 2 (20%) | 5 (25%) |
| T3 | 5 (50%) | 6 (60%) | 11 (55%) |
| T4 | 2 (20%) | 2 (20%) | 4 (20%) |
| Radiation treatment dose | | | |
| 60–64 Gy | 48 (84%) | 42 (72%) | 90 (78%) |
| 74 Gy[a] | 9 (16%) | 16 (28%) | 25 (22%) |
| Anterior field area (side of equivalent sq.) | | | |
| < 7.9 cm | 5 (9%) | 4 (7%) | 9 (8%) |
| 8–10.9 cm | 41 (72%) | 44 (76%) | 85 (74%) |
| 11 ≥ cm | 11 (19%) | 10 (17%) | 21 (18%) |

[a]The intervention group had a higher proportion of patients receiving 74 Gy treatment dosage, this was not statistically significant $\chi^2$ $P = 0.12$.

© 2001 Cancer Research Campaign per patient over the course of the 12 weeks of the study for both groups).

Contact through bleep calls to the nurse specialist was documented, using a proforma for recording information, this was collected over a period of 6 months. There were 24 uninitiated calls for a range of reasons. Advice that was requested was on a variety of concerns; 11 were primarily about managing cancer symptoms and 1 enquiry about blood tests. A further 12 calls were in relation to social difficulties, advice on other diseases and practical health care issues. Contact calls were not monitored for the control group.

### Comparison between intervention and control groups of symptoms and quality-of-life assessment at week 1

Patients completed the symptom assessments on the first clinic visit after beginning treatment, for 68% this was within the first week and for 91% this was within 13 days of starting radiotherapy. There were statistically significant differences in 7 of the symptom scores at week 1 between intervention and control groups, which we had not expected as the prediction would be that any benefits of intervention would be at their greatest when side effects from radiotherapy were at their maximum 6 weeks (Table 2). These differences were found in symptoms most commonly seen with prostate and bladder disease for example nocturia ($P < 0.006$), fatigue ($P < 0.04$) and impact on activity from bladder symptoms ($P < 0.01$). Men in the control group also had significantly more constipation ($P < 0.001$). The nurse specialist saw the men at start of therapy and the intervention focused on coping with urinary symptoms, provided practical advice and explored current concerns for those men commencing radiotherapy and this brief initial intervention is a possible explanation for the differences observed. Overall 52% (62% control group, 42% intervention group) of the men on self assessment of symptoms considered they had urinary symptoms that impacted on their daily activities to some degree. Data from the observer-rated RTOG scale indicated that only 28% of men had defined toxicity within the first week of treatment. Pre-treatment the RTOG scores showed no differences between intervention and control group. The RTOG assessment clearly focuses on selected aspects of toxicity, whilst the self-assessment questionnaire reflects patients' experience of symptoms, including whether symptoms pre-date, or are a consequence of, radiotherapy. There was no evidence of statistically significant differences in the EORTC QLQ30 at week 1 and both groups had high quality-of-life scores (Table 3).

### Comparison of symptoms and quality-of-life scores at weeks 3, 6 and 12

There were no significant differences in self-assessment scores at week 3, 6 and 12. The differences observed at week 1 had disappeared. The median score for urinary frequency in the intervention group (12) was higher at week 6 than in the control group (7), but not significantly so. A large amount of missing data at this time point prevented any conclusion being drawn about the longer-term benefits of nurse-led care (Table 4).

**Table 2** Assessment at week 1 for intervention and control groups for symptom self-assessment scale

| Measurements<br>Symptoms self assessment visual analogue scale | Randomisation | Patient numbers | Median | *IQR 25/75 | ** *P* |
|---|---|---|---|---|---|
| | | (Higher scores represent more severe symptoms) | | | |
| Urinary frequency | Intervention | 55 | 7 | 0/17 | 0.35 |
| | Control | 54 | 8 | 0/27 | |
| Urinary leakage | Intervention | 54 | 0 | 0/5 | 0.69 |
| | Control | 54 | 0 | 0/5 | |
| Nocturia | Intervention | 52 | 11 | 7/24 | 0.006 |
| | Control | 53 | 22 | 11/42 | |
| Pain with passing urine | Intervention | 56 | 0 | 0/3 | 0.07 |
| | Control | 54 | 0 | 0/13 | |
| Activity and bladder symptoms | Intervention | 57 | 0 | 0/3 | 0.01 |
| | Control | 56 | 5 | 0/20 | |
| Diarrhoea | Intervention | 56 | 0 | 0/5 | 0.43 |
| | Control | 53 | 0 | 0/7 | |
| Constipation | Intervention | 56 | 0 | 0/0 | 0.001 |
| | Control | 54 | 2 | 0/10 | |
| Cramp or abdominal pain | Intervention | 57 | 0 | 0/1 | 0.04 |
| | Control | 56 | 0 | 0/6 | |
| Sore anus | Intervention | 57 | 0 | 0/3 | 0.44 |
| | Control | 53 | 0 | 0/6 | |
| Activity and bowel symptoms | Intervention | 57 | 0 | 0/1 | 0.48 |
| | Control | 56 | 0 | 0/6 | |
| Fatigue | Intervention | 56 | 2 | 0/10 | 0.04 |
| | Control | 55 | 9 | 0/24 | |
| Sickness | Intervention | 57 | 0 | 0/0 | 0.01 |
| | Control | 55 | 0 | 0/2 | |
| Feeling unwell | Intervention | 57 | 0 | 0/3 | 0.01 |
| | Control | 56 | 3 | 0/14 | |

*IQR = interquartile range.**Mann–Whitney U tests.

 *© 2001 Cancer Research Campaign*

**Table 3** Data at week 1 for intervention and control groups for EORTC QLQ-C30 variables

| Measurements | Randomisation | Patient numbers | Median | *IQR 25/75 | ***P* |
|---|---|---|---|---|---|
| Functional scales | | (higher scores represent better functioning) | | | |
| Physical functioning | Intervention | 56 | 100 | 85/100 | 0.84 |
| | Control | 52 | 100 | 80/100 | |
| Role functioning | Intervention | 57 | 100 | 100/100 | 0.26 |
| | Control | 53 | 100 | 100/100 | |
| Emotional functioning | Intervention | 52 | 92 | 83/100 | 0.69 |
| | Control | 49 | 92 | 75/100 | |
| Cognitive functioning | Intervention | 54 | 100 | 83/100 | 0.96 |
| | Control | 52 | 100 | 71/100 | |
| Social functioning | Intervention | 56 | 100 | 83/100 | 0.67 |
| | Control | 53 | 100 | 83/100 | |
| **Global health status/QoL** | | | | | |
| Global quality of life | Intervention | 55 | 75 | 67/92 | 0.91 |
| | Control | 53 | 83 | 67/83 | |
| **Symptom scales/items** | | (higher scores represent more severe symptoms) | | | |
| Fatigue | Intervention | 55 | 22 | 0/33 | |
| | Control | 53 | 11 | 0/33 | 0.43 |
| Nausea and vomiting | Intervention | 57 | 0 | 0/0 | |
| | Control | 53 | 0 | 0/0 | 0.85 |
| Pain | Intervention | 55 | 0 | 0/17 | |
| | Control | 53 | 0 | 0/17 | 0.55 |
| Dyspnoea | Intervention | 57 | 0 | 0/0 | |
| | Control | 53 | 0 | 0/33 | 0.2 |
| Insomnia | Intervention | 57 | 0 | 0/50 | |
| | Control | 52 | 33 | 0/67 | 0.23 |
| Appetite loss | Intervention | 56 | 0 | 0/0 | |
| | Control | 53 | 0 | 0/0 | 0.06 |
| Constipation | Intervention | 57 | 0 | 0/0 | |
| | Control | 53 | 0 | 0/33 | 0.02 |
| Diarrhoea | Intervention | 55 | 0 | 0/0 | |
| | Control | 53 | 0 | 0/33 | 0.09 |
| Financial difficulties | Intervention | 56 | 0 | 0/0 | |
| | Control | 53 | 0 | 0/0 | 0.44 |

*IRQ = interquartile range.***P* = Mann–Whitney U test.

There were also no significant differences in RTOG scores between the intervention and control groups in terms of incidence of side-effects. Bowel symptoms rated grade 1 were experienced by 67% of the control group and 57% of the intervention group at 6 weeks. Only one man in the intervention group had toxicity of grade 2 or greater. Men in the control group experienced more severe bowel toxicity at 6 weeks, as defined by RTOG compared with those in the intervention group. No significant difference between groups was seen in maximum change in score from pre-radiotherapy ($\chi^2$ trend, $P = 0.4$) for both bowel (Figure 3) and bladder toxicitys (Figure 4).

Quality-of-life assessments at weeks 6 and 12 (Table 5) showed few differences between intervention and control groups. Functional scores were again high overall, with some evidence of significant difference between intervention and control groups in physical functioning at 12 weeks ($P = 0.05$), suggesting that those in the intervention arm were less physically impaired. Significantly higher levels of constipation were seen at this time point in the control group, compared with the intervention group ($P = 0.01$).

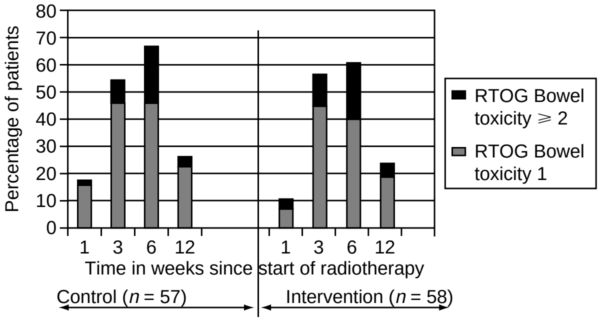

**Figure 3** Histogram comparing RTOG bowel toxicity between randomised groups. Maximum change in toxicity from pre radiotherapy: $p = 0.4$ ($\chi^2$ test for trend between groups)

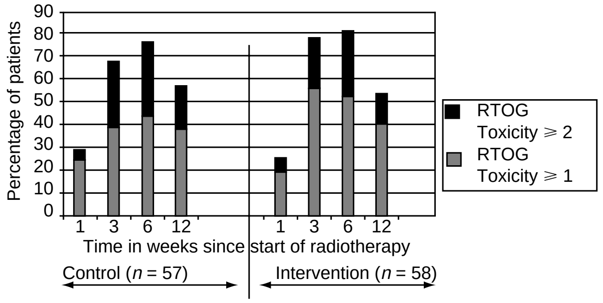

**Figure 4** Histogram comparing RTOG bladder toxicity between randomised groups. Maximum change in toxicity from pre radiotherapy: $p = 0.4$ ($\chi^2$ test for trend between groups)

© 2001 Cancer Research Campaign

**Table 4** Data at weeks 3, 6 and 12 for intervention and control groups for symptom assessment scale

| Symptoms self assessment visual analogue scale | Randomisation | Assessment at week 3 | | | | Assessment at week 6 | | | | Assessments at week 12 | | | |
|---|---|---|---|---|---|---|---|---|---|---|---|---|---|
| | | Patient numbers | Median | IQR 25/75 | ***P* | Patient numbers | Median | IQR 25/75 | ***P* | Patient numbers | Median | IQR 25/75 | ***P* |
| Urinary frequency | Intervention | 54 | 17.2 | 2/48 | 0.69 | 52 | 11.7 | 1/38 | 0.43 | 26 | 2 | 0/13 | 0.63 |
| | Control | 52 | 17 | 1/41 | | 53 | 7 | 0/39 | | 30 | 6 | 0/31 | |
| Urinary leakage | Intervention | 48 | 2 | 0/12 | 0.72 | 50 | 1 | 0/10 | 0.5 | 26 | 0 | 0/3 | 0.61 |
| | Control | 53 | 3 | 0/14 | | 52 | 1 | 0/17 | | 28 | 0 | 0/11 | |
| Nocturia | Intervention | 54 | 3 | 0/39 | 0.14 | 53 | 22 | 8/47 | 0.55 | 26 | 12 | 4/34 | 0.24 |
| | Control | 53 | 16 | 1/37 | | 52 | 26.7 | 9/60 | | 27 | 22.5 | 6/45 | |
| Pain with passing urine | Intervention | 52 | 6 | 0/26 | 0.79 | 52 | 4 | 0/27 | 0.95 | 26 | 0 | 0/7 | 0.43 |
| | Control | 52 | 4 | 0/28 | | 53 | 3 | 0/27 | | 28 | 3 | 0/11 | |
| Activity and bladder symptoms | Intervention | 54 | 14.5 | 0/39 | 0.87 | 54 | 6 | 0/32 | 0.73 | 27 | 1 | 0/19 | 0.37 |
| | Control | 54 | 13 | 1/39 | | 53 | 8 | 0/32 | | 30 | 7 | 0/31 | |
| Diarrhoea | Intervention | 54 | 8 | 0/28 | 0.76 | 51 | 3 | 0/14 | 0.37 | 26 | 0 | 0/7 | 0.23 |
| | Control | 54 | 7 | 2/24 | | 53 | 6 | 0/16 | | 30 | 3 | 0/18 | |
| Constipation | Intervention | 53 | 1 | 0/13 | 0.17 | 53 | 2 | 0/15 | 0.77 | 26 | 0 | 0/4 | 0.5 |
| | Control | 54 | 1 | 0/6 | | 52 | 1 | 0/14 | | 30 | 0 | 0/4 | |
| Cramp or abdominal pain | Intervention | 53 | 0 | 0/6 | 0.61 | 50 | 1 | 0/5 | 0.38 | 27 | 0 | 0/0 | 0.27 |
| | Control | 52 | 1 | 0/6 | | 52 | 0 | 0/2 | | 29 | 0 | 0/3 | |
| Sore anus | Intervention | 52 | 8 | 1/26 | 0.63 | 53 | 10 | 0/22 | 0.73 | 26 | 0 | 0/17 | 0.97 |
| | Control | 53 | 6 | 0/32 | | 51 | 7 | 0/32 | | 27 | 1 | 0/13 | |
| Activity and bowel symptoms | Intervention | 54 | 7 | 0/22 | 0.65 | 54 | 1 | 0/18 | 0.82 | 27 | 0 | 0/1 | 0.13 |
| | Control | 54 | 2 | 0/27 | | 53 | 1 | 0/22 | | 30 | 1 | 0/12 | |
| Fatigue | Intervention | 54 | 14.3 | 2/44 | 0.6 | 51 | 14.3 | 0/39 | 0.64 | 25 | 6 | 0/26 | 0.38 |
| | Control | 51 | 12 | 4/24 | | 53 | 12.7 | 4/25 | | 30 | 8 | 0/38 | |
| Sickness | Intervention | 52 | 0 | 0/2 | 0.99 | 51 | 0 | 0/3 | 0.41 | 26 | 0 | 0/0 | 0.19 |
| | Control | 51 | 0 | 0/1 | | 53 | 0 | 0/1 | | 29 | 0 | 0/1 | |
| Feeling Unwell | Intervention | 54 | 8 | 0/28 | 0.3 | 54 | 5 | 0/24 | 0.77 | 27 | 0 | 0/13 | 0.24 |
| | Control | 54 | 2 | 0/17 | | 53 | 4 | 0/15 | | 30 | 2 | 0/14 | |

*IQR = interquartile range.***P* = Mann–Whitney U tests.

© 2001 Cancer Research Campaign

**Table 5** Data at weeks 6 and 12 for intervention and control groups for quality of life variables

| Measurements | Randomisation | Assessment at week 6 | | | | Assessment at week 12 | | | |
|---|---|---|---|---|---|---|---|---|---|
| | | Patient numbers | Median | *IQR25/75 | ***P* | Patient numbers | Median | *IQR 25/75 | ***P* |
| **Functional scales** | | (higher scores represent better functioning) | | | | | | | |
| Physical functioning | Intervention | 53 | 100 | 80/100 | | 47 | 100 | 100/100 | |
| | Control | 44 | 100 | 80/100 | 0.49 | 43 | 100 | 80/100 | 0.05 |
| Role functioning | Intervention | 53 | 100 | 100/100 | | 48 | 100 | 100/100 | |
| | Control | 47 | 100 | 100/100 | 0.38 | 44 | 100 | 100/100 | 0.68 |
| Emotional functioning | Intervention | 54 | 92 | 75/100 | | 47 | 92 | 75/100 | |
| | Control | 43 | 92 | 67/100 | 0.45 | 43 | 83 | 67/100 | 0.24 |
| Cognitive functioning | Intervention | 52 | 100 | 67/100 | | 48 | 100 | 83/100 | |
| | Control | 44 | 92 | 67/100 | 0.84 | 41 | 83 | 83/100 | 0.89 |
| Social functioning | Intervention | 53 | 100 | 67/100 | | 47 | 100 | 83/100 | |
| | Control | 44 | 83 | 67/100 | 0.09 | 43 | 100 | 67/100 | 0.44 |
| **Global health status/QoL** | | | | | | | | | |
| Global quality of life | Intervention | 54 | 75 | 58/83 | | 48 | 79 | 67/83 | |
| | Control | 44 | 67 | 58/83 | 0.29 | 43 | 67 | 58/83 | 0.16 |
| **Symptom scales/Items** | | (higher scores represent more symptoms) | | | | | | | |
| Fatigue | Intervention | 51 | 22 | 0/44 | | 48 | 22 | 0/33 | |
| | Control | 46 | 33 | 11/44 | 0.7 | 44 | 22 | 0/33 | 0.85 |
| Nausea and vomiting | Intervention | 53 | 0 | 0/0 | | 46 | 0 | 0/0 | |
| | Control | 47 | 0 | 0/0 | 0.12 | 43 | 0 | 0/0 | 0.06 |
| Pain | Intervention | 50 | 0 | 0/16 | | 47 | 0 | 0/17 | |
| | Control | 46 | 0 | 0/21 | 0.45 | 45 | 17 | 0/17 | 0.36 |
| Dyspnoea | Intervention | 54 | 0 | 0/33 | | 48 | 0 | 0/33 | |
| | Control | 47 | 0 | 0/33 | 0.42 | 45 | 0 | 0/33 | 0.73 |
| Insomnia | Intervention | 53 | 33 | 0/67 | | 48 | 33 | 0/33 | |
| | Control | 47 | 33 | 0/67 | 0.91 | 45 | 33 | 0/33 | 0.91 |
| Appetite loss | Intervention | 54 | 0 | 0/0 | | 48 | 0 | 0/0 | |
| | Control | 47 | 0 | 0/0 | 0.38 | 45 | 0 | 0/0 | 0.71 |
| Constipation | Intervention | 53 | 0 | 0/33 | | 48 | 0 | 0/33 | |
| | Control | 46 | 0 | 0/33 | 0.06 | 45 | 33 | 0/33 | 0.01 |
| Diarrhoea | Intervention | 54 | 0 | 0/33 | | 46 | 0 | 0/33 | |
| | Control | 46 | 17 | 0/33 | 0.51 | 45 | 0 | 0/33 | 0.58 |
| Financial difficulties | Intervention | 54 | 0 | 0/0 | | 46 | 0 | 0/0 | |
| | Control | 44 | 0 | 0/0 | 0.02 | 43 | 0 | 0/0 | 0.68 |

*IRQ = interquartile range.** *P* = Mann–Whitney U tests.

### Comparison of symptoms over time

The time course of symptoms in the treatment group were compared using univariate and multivariate analysis to take account of variations in presenting factors.

The median of the maximum symptom score for each symptom over the time of radiotherapy treatment was used to define high and low symptom score groups of men. This created a dichotomous variable to compare men with high symptom scores with those with low symptom scores. Logistic regression was then used to model causative factors for high symptom scores.

In the univariate analysis, high symptom scores had no significant relationship with the randomised treatment group. In the multivariate analysis controlling for demographic, disease- and treatment-related factors there was no significant relationship between treatment group and symptom scores over the time of radiotherapy (Table 6).

### Patient satisfaction

Men were overall very content with their clinical care in both groups. However, those in the intervention group were significantly more satisfied ($P < 0.002$) with their follow-up care than those men attending the control group (Table 7). The nurse-led clinic was perceived as providing a greater amount of information: 91% (50) of the men in the intervention group were positive about this aspect, compared to 82% (42) in the control group. All patients rated the experience of radiotherapy treatment very positively, but in the control group 23% of men commented on the lack of continuity in follow-up care. Men in the intervention group felt well informed, felt their concerns were taken seriously, liked the continuity and the fact that their family was included in the consultations.

### Economic evaluation

The intervention provided cost benefits (Table 8). Service costs were significantly higher ($P < 0.001$) for those in the control group with a total cost saving of 31% (£10 548) in service costs, 38% (£344) in microbiology costs and 7% (£50) in the cost of medication. In total a 31% (£10 942) saving in health care costs were seen in the intervention group, compared with the control group. Service costs were lower in the intervention group, partly as the result of the nurse being cheaper to employ, as well as patients having less additional services. The cost of the nurse per clinic visit, was less (£71 for medical appointments compared with £52

© 2001 Cancer Research Campaign

**Table 6** Results of logistic regression analysis of high/low self assessed symptoms scores

| Factor | No. high symptom score% | Univariate analysis Odds ratio (OR) 95% CI | *P* | Multivariate analysis Odds ratio (OR) 95% CI | *P*[a] |
|---|---|---|---|---|---|
| Constipation | | | | | |
| Control | 26/56 (46.4) | 1 | | 1 | |
| Intervention | 32/58 (55.2) | 1.4 (0.6–2.9) | 0.35 | 2.5 (0.7–8.2) | 0.12 |
| Cramps | | | | | |
| Control | 24/55 (43.6) | 1 | | 1 | |
| Intervention | 26/58 (44.8) | 1.0 (4.9–2.2) | 0.89 | 1.9 (0.7–5.3) | 0.19 |
| Sore anus | | | | | |
| Control | 27/56 (48.2) | 1 | | 1 | |
| Intervention | 28/55 (50.9) | 1.11 (0.52–2.3) | 0.77 | 1.4 (0.59–3.73) | 0.4 |
| Diarrhoea | | | | | |
| Control | 28/56 (50) | 1 | | | |
| Intervention | 29/58 (50) | 1 (0.4–2.08) | 0.99 | 0.97 (0.3–2.4) | 0.96 |
| Fatigue | | | | | |
| Control | 25/56 (44.6) | 1 | | 1 | |
| Intervention | 32/58 (55.2) | 1.52 (0.72–3.1) | 0.26 | 1.7 (0.7–4.42) | 0.21 |
| Urinary frequency | | | | | |
| Control | 27/55 (49.1) | 1 | | | |
| Intervention | 28/58 (48.3) | 0.9 (0.46–2.02) | 0.93 | 1.10 (0.45–2.64) | 0.82 |
| Leakage | | | | | |
| Control | 30/56 (53.6) | 1 | | 1 | |
| Intervention | 26/58 (44.8) | 0.7 (0.33–1.47) | 0.35 | 1.0 (0.4–2.44) | 0.99 |
| Nocturia | | | | | |
| Control | 29/56 (51.8) | 1 | | | |
| Intervention | 28/58 (48.3) | 0.8 (0.4–1.81) | 0.7 | 0.9 (0.37–2.15) | 0.81 |
| Pain when passing urine | | | | | |
| Control | 30/56 (53.6) | 1 | | | |
| Intervention | 26/58 (44.8) | 0.7 (0.33–1.47) | 0.35 | 0.88 (0.37–2.05) | 0.77 |

[a]Multiple regression controlling for demographic and treatment variables.

**Table 7** Frequency data from survey of satisfaction with care

| **Frequency data from survey of satisfaction with care** | | | | | | | | | |
|---|---|---|---|---|---|---|---|---|---|
| **Question** | | **Number of patients** | **Median** | **Interquartile range** | ***P**** | | | | |
| **Satisfaction with medical and nursing care** | | | | | | | | | |
| Global score | Intervention | 55 | 97.5 | 82.5/100 | < 0.002 | | | | |
| | Control | 52 | 85 | 68.1/96.6 | | | | | |
| **Question: How did you feel about** | | **Patient numbers** | **Negative response** | **Percentage** | **Neutral response** | **Percentage** | **Positive response** | **Percentage** | |
| 1. The technical aspects of treatment | Intervention | 55 | 0 | 0% | 2 | 4% | 53 | 96% | |
| | Control | 52 | 0 | 0% | 3 | 6% | 49 | 94% | |
| 2. The amount of information you were given | Intervention | 55 | 5 | 9% | 0 | 0% | 50 | 91% | |
| | Control | 51 | 4 | 8% | 5 | 10% | 42 | 82% | |
| 3. Your worries and concerns were taken seriously | Intervention | 54 | 0 | 0% | 3 | 6% | 51 | 94% | |
| | Control | 52 | 1 | 2% | 8 | 15% | 43 | 83% | |
| 4. That checks were made to see if you were okay | Intervention | 55 | 0 | 0% | 2 | 4% | 53 | 96% | |
| | Control | 52 | 2 | 4% | 5 | 10% | 45 | 86% | |
| 5. The way things were explained to you | Intervention | 55 | 0 | 0% | 2 | 4% | 53 | 96% | |
| | Control | 51 | 1 | 2% | 6 | 12% | 44 | 86% | |
| 6. That your family were considered | Intervention | 55 | 3 | 5% | 6 | 11% | 46 | 84% | |
| | Control | 50 | 4 | 8% | 10 | 20% | 36 | 72% | |
| 7. The amount that was known about your case | Intervention | 55 | 0 | 0% | 4 | 7% | 51 | 93% | |
| | Control | 51 | 1 | 2% | 9 | 18% | 41 | 80% | |
| 8. That you knew who was looking after you | Intervention | 55 | 1 | 2% | 3 | 5% | 51 | 93% | |
| | Control | 52 | 8 | 15% | 10 | 19% | 34 | 65% | |
| 9. That your symptoms were managed as best they could | Intervention | 55 | 0 | 0% | 2 | 4% | 53 | 96% | |
| | Control | 51 | 1 | 2% | 3 | 6% | 47 | 92% | |
| 10. The awareness of your needs | Intervention | 55 | 1 | 2% | 2 | 4% | 52 | 94% | |
| | Control | 51 | 3 | 6% | 4 | 8% | 44 | 86% | |

*Mann–Whitney U test.

 *© 2001 Cancer Research Campaign*

**Table 8** Cost savings between randomised groups groups

| | Service costs | Microbiology | Medication | Total costs |
|---|---|---|---|---|
| Control (*n* = 57) | £33 650 | £901 | £755 | £35 306 |
| Intervention (*n* = 58) | £23 102 | £557 | £704 | £24 363 |
| Total | £56 751 | £1458 | £1460 | £59 668 |
| Saving | £10 548 | £345 | £51 | £10 943 |
| Percentage saving | 31% | 38% | 7% | 31% |
| **P* | 0 | 0.01 | 0.87 | 0 |

**P* = Mann–Whitney U test.

**Table 9** Service costs

| | Randomised group | Median | IQR range* | Sum cost | All | *P** |
|---|---|---|---|---|---|---|
| Overnight stays | Intervention | £0 | 0–0 | £3005 | | 0.31 |
| (Cost £250.44) | Control | £0 | 0–0 | £2254 | £5259 | |
| Day case | Intervention | £0 | 0–0 | $370 | | 0.30 |
| (Cost £185) | Control | £0 | 0–0 | £925 | £1295 | |
| Outpatient appointments | Intervention | £281 | £228–351 | £16 059 | | 0.001 |
| (Cost**) | Control | £426 | £355–487 | £24 867 | £40 926 | |
| Telephone contact | Intervention | | | £54 | | |
| (Cost for 2 calls £0.93p) | Control | | | £0 | £54 | |
| Radiotherapy nurse | Intervention | £52 | £0–105 | £3614 | | 0.70 |
| (Cost £52.37p) | Control | £52 | £0–105 | £5604 | £9217 | |
| Total service costs | Intervention | | | £23 102 | | 0.001 |
| | Control | | | £33 650 | £56 751 | |

*Mann–Whitney U test. **Cost for Dr £71.06 and for nurse £52.37.

for the nurse-led follow-up), but also in the intervention there was reduced outpatient attendance (Table 9). These overall and service cost savings would only be realised if the nurse-led service was provided as a component of existing care or by re-allocating existing health-care resources.

The median cost of outpatient attendance per patient was £280 in the intervention group, with a range of £288–351, whilst in the control group the median cost per patient was £426, with a range of £355–487. The reason for this was the smaller number of clinic appointments in the intervention group. Overall medication and investigations such as microbiology accounted for only a small proportion of the economic costs, and it was the changes to the way that outpatient care was managed that reduced the health-care costs overall. Replacing routine outpatient appointments with telephone contact was cheaper.

### DISCUSSION

This study was designed to evaluate a nurse-led clinic not only in patient outcomes such as quality of life, toxicity, self-assessed symptoms but also its effect on service provision. Statistically significant differences were seen between the two-randomised groups in symptoms within the first week of radiotherapy treatment. This diminished over the course of radiotherapy and over time there was little evidence of differences between the 2 groups. The differences seen in the first week of radiotherapy are unlikely to be due to bias in patient allocation because of the randomised nature of the study and similar demographic and treatment characteristics between the 2 groups. We believe that the brief intervention at the start of radiotherapy by the nurse in discussing symptoms, providing information and practical strategies for support was helpful and secured these advantages. Further work in exploring the benefits of this intervention are required.

The hypothesis of this study was based on the premise that the intervention would be able to influence urinary symptoms and quality of life at 6 weeks. At the time of setting up of this study little was known as to the extent of self-assessed morbidity in men with prostate cancer. There has been in recent years a growing body of literature exploring prostate cancer and its effect on men's quality of life. These studies have identified that men have high quality of life and that the conventional global quality-of-life assessments are insensitive in this patient population (Kemmler et al, 1999). However, although we were unable to demonstrate a difference in radiotherapy morbidity the study provides useful data in evaluating such health-care change. Over 50% of the patients in the first week of radiotherapy had disease-related symptoms, such as urinary frequency or nocturia, which were felt to impact on daily activities. Much of the intervention work was in mediating the effect of these rather than the acute side effects that were initially the focus of this study. This is important to remember when considering the benefits of this approach and the training or skills of the practitioner involved in monitoring patients. Many centres have developed innovative roles for therapeutic radiographers, whose skills may be different in relation to that of the nurse in managing disease-related symptoms. This was demonstrated in that 50% of the queries from bleep contact were not related to radiotherapy side effects but more general health-related concerns. This should be considered when developing innovative roles in that side effects arise for patients within the context of the wider cancer experience. Comparison, between the 2 randomised groups, in the severity of acute side effects showed few differences. Interventions to alleviate or mediate acute side effects have

*© 2001 Cancer Research Campaign* 

focused on cystitis and diarrhoea. However, in this study, nocturia and fatigue were 2 of the most troubling symptoms that impacted on physical functioning.

The issue of autonomy is often raised as a potential barrier to the development of nursing roles, but this study was designed and co-ordinated in collaboration with medical colleagues. Patients receiving treatment were discussed and their treatment audited on a weekly basis by the multidisciplinary team. The consultant therefore delegated the day-to-day management of the patients' care to the specialist nurse. The model explored within this study was of 'nurse-led' care rather than 'nurse-alone' care, which is an important distinction as the support of the multidisciplinary team is essential to be able to function in such a role. Patients commented that one of the benefits of nurse-led care was the continuity of care. Translating this service into a wider patient population would increase the caseload of the nurse, however the fundamental change away from routine appointments and the use of telephone contact could reduce workload. However, some cancer patient groups may not be suitable for nurse-led care, for example men with bladder cancer required more medical services and a wider extent of medication than men with prostate cancer. Eligibility criteria would help in determining those most suitable for nurse-led care.

The number of statistical tests used when evaluating differences between groups across the various measures must be considered and thus 'borderline' $P$ values (close to the conventional $P = 0.05$ level) should be interpreted with caution. The study was not designed to consider subtle differences and patient numbers would have to increase substantially in order to detect small differences with an acceptable level of power.

The secondary hypothesis was confirmed in that nurse-led care provided cost savings and patients were more satisfied with health care. Positive benefits were identified in the evaluation of nurse-led care. Overall during the course of the study there was an approximate cost saving of 31%. The introduction of specialist nursing care in other health-care areas such as paediatrics (Brooten et al, 1986) or mental illness (Brooker and Buuterworth, 1991) has previously identified cost savings. Previous studies have identified that nursing care is cheaper than medical care but they have not taken this forward into realising the savings by reducing medical staffing levels and transferring resources to nursing posts. In this study the cost savings were due in main to changes in the way services were delivered, reducing both outpatient appointments and inpatient stays. The nurse-led service was placed as part of existing outpatient services.

The ability to generalise these cost savings into wider practice settings would require not only a shift in how care was organised but a movement of resources. The methods of working out the economics of health care are inevitably an oversimplification as the units of analysis used by health-care services in costing elements of care are rarely based on true financial cost. However, what this study offers is a comparison of how a change in radiotherapy follow-up can be reflected in the utilisation of hospital services. The existing divisions in radiotherapy care, funding systems and available management of resources all combine to impede changes in practice. If funding came as a single element for supportive care rather than specific posts then care could be organised more efficiently according to the most appropriate person to deliver that care. This could be a specialist nurse, therapy radiographer or physician. To realise these economic benefits a movement towards a more patient-centred system is required.

### ACKNOWLEDGEMENTS

I would like to acknowledge the Cancer Research Campaign for providing a Nursing Clinical Research Fellowship to support this work. The Institute of Cancer Research also supported this work. Clinical work was undertaken at The Royal Marsden NHS Trust who receive a proportion of its funding from the NHS Executive; the views expressed in this publication are those of the authors and not necessarily those of the NHS executive.

### REFERENCES

Aaronson N, Ahmedzai B, Bergman M, Bullinger A, Filbiberti H, Fletcher S, de Haes S, Klee D, Osoba D, Razavi B, Rofe S, Schraub K, Sneeuw M, Sullivan M and Takeda F (1993) The European Organisation for Research and treatment of cancer QLQ-C30: A quality of life instrument for use in international clinical trials in Oncology. *J Natl Cancer Inst* **85**(**5**): 365–376

Brooker C and Butterworth T (1991) Working with families caring for a relative with schizophrenia: the evolving role of the community psychiatric nurse. *Int J Nurs Stud* **28**(**2**): 189–200

Brooten D, Kumar S, Brown L, Butts P, Finkler S, Bakewell-Sachs S, Gibbons A and Delivoria-Papadopoulos (1986) A randomised clinical trial of early hospital discharge and home follow-up of very low birth weight infants. *New Engl J Med* **315**(**15**): 934–939

Brown S and Grimes D (1993) 'A meta-analysis of clinical care; clinical outcomes and cost effectiveness of nurses in primary care roles, nurse practitioners and certified nurse midwives'. Washington DC, American Nurses Association

Campbell J, German L and Dodwell D (2000) Radiotherapy outpatient review: A nurse-led clinic. *Clin Oncol* **12**: 104–107

Cox J, Stetz J and Pajak T (1995) Toxicity criteria of the radiation therapy oncology group (RTOG) and the European Organization for research and treatment of cancer (EORTC). *Int J Radiat Oncol Biol Phys* **31**(**5**): 1341–1346

Faithfull S (1995) Just grin and bear it and hope that it will go away: coping with urinary symptoms from pelvic radiotherapy. *Euro J Cancer Care* **4**: 158–165

James N, Guerrero D and Brada M (1994) Who should follow up cancer patients? Nurse specialist based outpatient care and the introduction of a phone clinic system. *Clin Oncol* **6**: 283–287

Kemmler G, Holzner B and Kopp M (1999) Comparison of two quality of life instruments for cancer patients: the Functional Assessment of Cancer Therapy-General and the European Organization for Research and Treatment of Cancer quality of life questionnaire-C30. *J Clin Oncol* **17**: 2932–2940

Mathews J, Altman D, Campbell M and Royston P (1990) Analysis of serial measurements in medical research. *Brit Med J* **300**: 230–235

Norcross Weintraub F and Hagopian A (1990) The effect of nursing consultation on anxiety, side-effects, and self care of patients receiving radiation therapy. *Oncol Nurs Forum* **17**(**3**): 31–38

Richardson G and Maynard A (1995) 'Fewer doctors? More nurses? A review of the knowledge base of doctor-nurse substitution', York, University of York

Ridsdale L, Robins D, Cryer C and Williams H (1997) Feasability and effect of nurse run clinics for patients with epilepsy in general practice: randomised controlled trial. *Brit Med J* **314**: 120–122

Rotman M, Rogow L, DeLeon G and Heskel N (1977) Supportive therapy in radiation oncology. *Cancer* **39**: 744–750

Steinberg M and Rose C (1996) Posttreatment follow-up of radiation oncology patients in a managed care environment. *Int J Radiat Oncol Biol Phys* **35**(**1**): 113–116

Strohl R (1988) The nursing role in radiation oncology symptom management of acute and chronic reactions. *Oncol Nurs Forum* **15**(**4**): 429–434

Thomas L and MacMillan J (1995) 'Obtaining patients' views of nursing care to inform the development of a patient satisfaction scale. *Intern J Qual Health Care* **2**(**2**): 153–163

Yates B (1998) Formative evaluation of costs, cost-effectiveness, and cost benefit: toward cost -procedures-process, *in* Bickman L and Rog D (eds.), *Handbook of applied social research methods* London, Sage publications

 *© 2001 Cancer Research Campaign*

## References

[PDF_00999] Aaronson N. K., Ahmedzai S., Bergman B., Bullinger M., Cull A., Duez N. J., Filiberti A., Flechtner H., Fleishman S. B., de Haes J. C. (1993). The European Organization for Research and Treatment of Cancer QLQ-C30: a quality-of-life instrument for use in international clinical trials in oncology.. J Natl Cancer Inst.

[PDF_01004] Brooker C., Butterworth C. (1991). Working with families caring for a relative with schizophrenia: the evolving role of the community psychiatric nurse.. Int J Nurs Stud.

[PDF_01007] Brooten D., Kumar S., Brown L. P., Butts P., Finkler S. A., Bakewell-Sachs S., Gibbons A., Delivoria-Papadopoulos M. (1986). A randomized clinical trial of early hospital discharge and home follow-up of very-low-birth-weight infants.. N Engl J Med.

[PDF_01016] Cox J. D., Stetz J., Pajak T. F. (1995). Toxicity criteria of the Radiation Therapy Oncology Group (RTOG) and the European Organization for Research and Treatment of Cancer (EORTC). Int J Radiat Oncol Biol Phys.

[PDF_01019] Faithfull S. (1995). 'Just grin and bear it and hope that it will go away': coping with urinary symptoms from pelvic radiotherapy.. Eur J Cancer Care (Engl).

[PDF_01022] James N. D., Guerrero D., Brada M. (1994). Who should follow up cancer patients? Nurse specialist based outpatient care and the introduction of a phone clinic system.. Clin Oncol (R Coll Radiol).

[PDF_01025] Kemmler G., Holzner B., Kopp M., Dünser M., Margreiter R., Greil R., Sperner-Unterweger B. (1999). Comparison of two quality-of-life instruments for cancer patients: the functional assessment of cancer therapy-general and the European Organization for Research and Treatment of Cancer Quality of Life Questionnaire-C30.. J Clin Oncol.

[PDF_01029] Matthews J. N., Altman D. G., Campbell M. J., Royston P. (1990). Analysis of serial measurements in medical research.. BMJ.

[PDF_01039] Rotman M., Rogow L., DeLeon G., Heskel N. (1977). Supportive therapy in radiation oncology.. Cancer.

[PDF_01041] Steinberg M. L., Rose C. M. (1996). Posttreatment follow-up of radiation oncology patients in a managed care environment.. Int J Radiat Oncol Biol Phys.

[PDF_01044] Strohl R. A. (1988). The nursing role in radiation oncology: symptom management of acute and chronic reactions.. Oncol Nurs Forum.

[PDF_01046] Thomas L. H., MacMillan J., McColl E., Priest J., Hale C., Bond S. (1995). Obtaining patients' views of nursing care to inform the development of a patient satisfaction scale.. Int J Qual Health Care.

